# The Population Health Research Network - Population Data Centre Profile

**DOI:** 10.23889/ijpds.v4i2.1130

**Published:** 2019-11-20

**Authors:** F Flack, M Smith

**Affiliations:** 1 Population Health Research Network, University of Western Australia

## Abstract

The Population Health Research Network (PHRN) is an Australian data linkage infrastructure capable of securely and safely linking and integrating data collections from a wide range of sources. It is an example of a national data linkage infrastructure in a country with a federated system of government. This population data centre profile describes Australia’s unique approach to enabling access to linked data from single jurisdictions and from multiple jurisdictions. It covers the background to the establishment of the PHRN as well as information about how it operates today including operating models, governance, data, data linkage and data access. Some of the challenges of data linkage across jurisdictions are also discussed.

## Background

Australia has a long history of using linked administrative data for research which dates back to the 1960’s when Professor Michael Hobbs was instrumental in the establishment of population-based data linkage in Australia [1]. His work laid the foundations for the establishment of systematic data linkage in Western Australia (WA), and the establishment of the WA Data Linkage Branch in 1995 [1]. The New South Wales (NSW) and Australian Capital Territory (ACT) data linkage unit, the Centre for Health Record Linkage (CHeReL), was established in 2006 [2]. The Australian Institute for Health and Welfare (AIHW) also linked data from a range of administrative data collections including the National Death Index and Australian Cancer Database for its own work and on behalf of clients.

A number of factors influenced the approach Australia has taken to linked administrative data including:

- The success of the WA Data Linkage Branch and NSW Centre for Health Record Linkage
- The Australian system of government which is a federation of six states and two self-governing territories.
- The shared responsibility for running the Australian health system by the Australian, state and territory governments results in health and human services data collections distributed across jurisdictions.

These factors led to the recognition that every jurisdiction should have data linkage capacity and there should also be the capability to link data across jurisdictions. In Australia this is called cross-jurisdictional data linkage and it involves bringing together information, from different sources in different jurisdictions, but relating to the same individual. This is necessary, because some data is collected by state and territory governments e.g. hospital data and some is collected by the Australian Government e.g. prescribing data.

This opportunity for Australian research was recognised in the Australian Government’s National Collaborative Research Infrastructure Strategy Strategic Roadmap 2006 [3]. After extensive national consultation an investment plan was agreed and funding from the National Collaborative Research Infrastructure Strategy was allocated to establish the Population Health Research Network (PHRN) in 2009 with co-funding from state and territory governments and academic institutions. The funding model has worked well and is continuing.

The original objective of the PHRN was to build a nationwide data linkage infrastructure capable of securely and safely linking and integrating data collections from a wide range of sources. This infrastructure is now in place and PHRN continues to provide coordination, funding and other support for its network of data centres across Australia.

## Approach

### Population setting

The PHRN is a national network of data centres which links data collected in all Australian jurisdictions on all Australians. Australia is a federation of six states and two self-governing territories which together make up the Commonwealth of Australia. The Australian health system is jointly run by all levels of government – federal, state and territory and local. There is also a significant private health system in Australia. This means that health data is collected by different jurisdictions. For example, hospital data is collected by state and territory governments and pharmaceutical benefits data is collected by the federal government. The population of Australia is currently approximately 25 million.

### Operating model

The PHRN is primarily a distributed model where data is managed at the jurisdictional level. There are two layers of distribution. The operating models used by PHRN affiliated data centres are at least partially based on the model described by Kelman and colleagues [4]. In this model identifiers such as name, address and date of birth are separated from content data e.g. diagnosis, and provided to a specialist data centre who uses this information to create a map or index of all the individuals in the population and where their content data is stored. The content data remains with the original data custodians and is only brought together for specific approved projects. This is a distributed model as the content data is not stored in the same location as the identifiers and linkage keys.

**Terminology:** Note that in Australia, the units creating the linkage maps/indexes and coordinating access to linked data are generally known as data linkage units. For the purposes of this paper, they are referred to as data centres.

Many jurisdictions are now incorporating aspects of a repository model into their operations. In general, this involves the creation of an integrated repository of content data from the most commonly linked data collections [5, 6]. The impetus to shift to this combination model is increased efficiency of data extraction. Decisions about the use of the content data generally remain with the individual data custodians.

The second level of distribution in the PHRN is that there is not a single data centre for the nation. Each state and territory is serviced by a data centre and there is also a national data centre at the AIHW. For cross-jurisdictional projects one or more of the data centres may be involved depending on the study design and legal and policy limitations.

To assist readers to understand the different functions of the data centres in the PHRN the following three broad functions are defined:

Data linkage – a method of determining whether information derived from different sources is related to the same individual or event. This is usually done using identifiable linkage variables such as name, address, sex and date of birth with a probabilistic matching technique.

Data repository - a large database infrastructure where linkable content data i.e. data without overt identifiers such as names and addresses from multiple sources is brought together to be managed, stored and provided to an approved access environment for approved research projects.

Secure access environment – a specialised environment either physical or virtual where sensitive data can be safely accessed and analysed. Sometimes called a safe haven. The data centres which are part of the PHRN are shown in Table 1 below. The two territories (Australian Capital Territory and the Northern Territory) do not operate their own data centre and obtain data linkage services from an adjacent state.

**Table 1: PHRN Affiliated Data Centres:** 

Data Centre	Jurisdictions	Data Linkage Unit	Data Repository	Safe Haven/Data Access	URL

CHeReL	Australian Capital Territory & New South Wales	Yes	Yes	No	http://www.cherel.org.au/
SANT DataLink	Northern Territory & South Australia	Yes	Yes	No	https://www.santdatalink.org.au/
Data Linkage Queensland	Queensland	Yes	No	No	https://www.health.qld.gov.au/hsu/link/datalink
Tasmanian Data Linkage Unit	Tasmania	Yes	No	No	http://www.menzies.utas.edu.au/research/research-centres/data-linkage-unit
Centre for Victorian Data Linkage	Victoria	Yes	Yes	No	https://www2.health.vic.gov.au/about/reporting-planning-data/the-centre-for-victorian-data-linkage
WA Data Linkage Branch	Western Australia	Yes	Yes	No	https://www.datalinkage-wa.org.au/
AIHW	Australian Government	Yes	Yes	Yes	https://www.aihw.gov.au/our-services/data-linkage
Secure Unified Research Environment	All	No	No	Yes	https://www.saxinstitute.org.au/our-work/sure/

For research applications involving data from more than one Australian jurisdiction the PHRN provides an online application form and a coordinated application and approval process to assist researchers to navigate the complex and varied approval processes in different jurisdictions. This process includes designing and assessing the feasibility of methods of achieving cross-jurisdictional linkage i.e. bring together data from different jurisdictions about the same individual. Client services officers from all the data centres involved in the project work together to coordinate, monitor and provide feedback on the progress of the application.

Other functions of the PHRN include:

- The provision of publically available information about research conducted using linked data e.g. website and webinars
- Information and training for researchers on what data is available and how to access it
- Training e.g. consumer involvement training for data centres, ethics training for human research ethics committees and client services training for client services officers
- Networking opportunities for researchers and data centre staff
- Advocacy for data linkage in Australia

### Architecture and information technology

The PHRN does not have standard IT platforms or technology for data linkage, data repositories or secure data access. Each PHRN affiliated data centre is able to choose the architecture and technology best suited to their needs. This means that there is a range of platforms and technologies used across the network.

The Online Application System is used for cross-jurisdictional data applications and is available to all jurisdictions to use for single jurisdiction applications if they choose.

### Governance, legislation and management

The PHRN is a national collaborative network. It is not an independent organisation. The University of Western Australia (UWA) is the PHRN lead agent and signatory to the head funding agreement with the Australian Government. UWA is therefore responsible for ensuring that the terms of the agreement with the Australian Government are met. The PHRN Program Office at UWA manages and coordinates the network. UWA contracts with PHRN Participants across Australia. The PHRN provides funding to all the data centres in the Network. The percentage of each data centre’s budget and the specific items that are covered by the PHRN depends on the strategic priorities of the PHRN and the amount of co-funding available to the data centre from state/territory governments, academic partners and cost recovery from users. The PHRN-related roles and responsibilities of each of the participating organisations are clearly defined and described in these contracts.

The PHRN Board is an independent advisory board which provides oversight and strategic direction for the PHRN [7]. The PHRN Board recommends funding allocation in line with the PHRN’s strategic directions. It receives advice from the PHRN Participant Council on strategy, policy, funding priorities, stakeholder engagement, performance and accountability [8]. The PHRN Participant Council is made up of one nominee from each of the PHRN data centres. The terms of reference allow for additional members who make significant contributions to the PHRN.

Each individual PHRN Participant has its own governance requirements depending on the type of organisation e.g. government, academic. The PHRN provides advice, guidance and coordination. However, each PHRN Participant must also operate within the legislative and policy requirements of the jurisdictions and organisations in which they operate.

PHRN contracts bind each organisation to comply with the Information Privacy Principles in *The Privacy Act* 1988 (Commonwealth) as well as with the privacy and other legislation in their own state or territory and all PHRN policies.

In addition to organisational contracts, data linkage staff with access to personal information and researchers who are provided with linked data are required to sign confidentiality agreements. These are legally binding contracts that outline the relevant legislation that applies to the data, the terms and conditions of its use and consequences of data misuse and privacy breaches.

The ethics and legal frameworks in which the PHRN operates are complex [9]. This complexity increases with number of data collections linked and number of jurisdictions from which the data is sourced.

Community involvement has been an important component in the success of the PHRN. At the Network level there have always been consumer representatives on governance committees. In addition, training in community involvement was offered to all PHRN data centres in the early years of the PHRN’s development. The provision of appropriate information about how data is linked and the kind of research conducted remains a priority for PHRN communications. Each individual PHRN Participant has involved the community in different ways including:

- Representation on governance committees
- Community advisory groups
- Community events e.g. community conversations
- Information on websites and social media

### Consent model

In Australia most privacy legislation (state/territory and Commonwealth) and the National Statement on Ethical Conduct in Human Research allow for the use of data without consent if certain conditions are met [10]. All PHRN data centres were established on the basis of a waiver of consent consistent with local ethics and legislative requirements.

Each data centre conducts routine linkage of a set of core data collections [11]. A waiver of consent for each of these data collections is required. Data centres also perform ad hoc linkages on request and these linkages may be conducted with consent, using an opt-out approach or with a waiver of consent.

In the early years of the PHRN it was recognised that the establishment of data centres in all jurisdictions would require human research ethics committees (HRECs) in all jurisdictions to understand data linkage and the ethical issues that arise from the use of linked data, particularly without consent. The PHRN developed a training workshop for HRECs which was run across the country [12].

### Privacy by design

The protection of privacy is of the highest importance to all PHRN affiliated data centres. Each data centre is responsible for implementing the most appropriate privacy and security measures for their data centre depending on their organisational, legislative and technological requirements.

The PHRN has an Information Governance Framework1The PHRN Information Governance Framework is for internal PHRN use only. It is not a publically available document which provides the PHRN data centres with guiding principles on how to implement privacy by design and is consistent with relevant international and national security standards for information security, privacy and risk management. The Framework covers the following areas of data centre operations:

Governance

- Risk Management
- Information Governance Policies
- Awareness, Education and Training
- Workforce Management
- Managing Third Parties
- Compliance

Operations

- Asset Management
- Information Exchange
- Physical Security
- Incident & Data Breach Management
- Resilience

Technology

- Operational Controls
- Access Control
- Mobile Devices
- Software Development and Acquisition
- Cryptography

Researchers accessing linked data through one of the PHRN data centres will receive unit record data (data at the person level) but will not gain access to names and addresses or other overt identifiers. They will only receive the minimum number of variables required to answer their specific research question. The extent of confidentialisation techniques used are determined on a case by case basis depending on the risk benefit ratio assessed by the ethics committee and data custodians e.g. age may be provided instead of date of birth.

#### Data linkage

Most PHRN DLUs use a probabilistic linkage technique but this may be in combination with a deterministic approach [13, 14]. The different data centres use different data linkage software. Some is commercially available such as LinkageWiz and or open source software such as ChoiceMaker. Other units have bespoke systems.

### Data linkage keys

The PHRN model is that a specialist data linkage unit generates the linkage keys. There is generally one linkage unit (data centre) which serves each Australian state and territory (see above). The AIHW is the data centre for Commonwealth data. Cross jurisdictional linkage is achieved by state data centres and AIHW working together.

Data linkage keys are updated at different times depending on the data centre and the data collection. The frequency of update can range from near real time to annually.

### Data sources

At the time of writing the following administrative data collections were routinely linked by all of the state/territory data centres in the PHRN. These data collections are collected at the state/territory population level. For most data collections, data is available for at least the last 10 years. Some collections go back several decades.

- Admitted patients (public hospitals)
- Births
- Cancer registry
- Deaths
- Perinatal
- Emergency Department (public hospitals)

More information about routinely linked data collections can be found at https://www.phrn.org.au/for-researchers/data-collections-available/.

Each data centre also routinely links a range of other data collections including other health data collections and human services and education data. The frequency of linkage/update of each data collection can vary from near real time to annually.

The AIHW links a number of Commonwealth data collections which have national coverage including the Medicare Benefits Scheme and Pharmaceutical Benefits Scheme data.

Most of the PHRN data centres do not provide standardised data products. Project specific datasets of unit record data are provided after the required approvals have been received.

### Data access

Access to linked data in Australia for research requires approval from each data custodian and at least one human research ethics committee. The relevant data centre also needs to agree that the project is technically feasible. The process to apply for data custodian approval for a project using data from a single jurisdiction differs between jurisdictions. Cross-jurisdictional data applications can be submitted on a national online application form [15]. Application for ethics approval is a separate process and may require applications to human research ethics committees in each jurisdiction from which the data is sourced. There has been some progress towards mutual acceptance of ethics approval between jurisdictions.

If the data custodians and human research ethics committee approve, the linked dataset can be provided to the researcher’s own analysis environment e.g. at their university. Currently most access to linked data occurs in this way. Some data custodians, often Commonwealth data custodians, will require the data to be accessed in a secure remote access facility (safe haven) such as the PHRN supported Secure Unified Research Environment facility (SURE) [16]. All files uploaded or downloaded from SURE are curated to ensure only approved data enters and exits the environment. There is also a secure physical data laboratory at the AIHW.

If the data is provided to the researcher’s own analysis environment they provide their own analysis software. A range of software is available in SURE including R, SAS, SPSS and STATA. In addition, researchers can bring their own software into SURE but this may incur additional costs.

### Noteworthy outputs

The investment in data linkage infrastructure in Australia has resulted in a significant increase in the number of research publications involving the use of linked data [17]. Noteworthy examples of research involving cross-jurisdictional, multi-jurisdictional and single jurisdiction linkage are listed below.

1. Gidding HF, Sheridan S, Fathima P, et al. Impact of childhood pneumococcal conjugate vaccine on nonnotified clinically suspected invasive pneumococcal disease in Australia. Paed Infect Dis J. 2019; https://doi.org/10.1097/INF.0000000000002314
  This article is a good example of how the PHRN links information from different data collections in different jurisdictions, but relating to the same individual. Data from three jurisdictions (Commonwealth, New South Wales and Western Australia) were linked through the collaboration of three data centres (AIHW, Centre for Health Record Linkage and Western Australian Data Linkage Branch) and made available to the research team through a secure remote access facility (SURE).
2. Mitchell RJ, Cameron CM, McClure R. Patterns of health care use of injured adults: A population-based matched cohort study. Injury. 2017 Jul;48(7):1393-1399. https://doi.org/10.1016/j.injury.2017.04.014.
  This article is a good example of multi-jurisdictional linkage where linked data from three jurisdictions (New South Wales, South Australia and Queensland) were provided to the research team for analysis. In this example there were no cross-border linkages. The data centre in each jurisdiction (CHeReL, SANT DataLink and Data Linkage Queensland) linked the data collections from their jurisdiction and used the state electoral roll to select a control cohort.
3. Crowe E, Pandeya N, Brotherton JML, et al. Effectiveness of quadrivalent human papillomavirus vaccine for the prevention of cervical abnormalities: Case-control study nested within a population based screening programme in Australia. BMJ (Online). 2014; 348, art. no. g1458. https://doi.org/10.1136/bmj.g1458
  This highly cited publication demonstrates the linkage of data collections from a single jurisdiction (Queensland) by the jurisdictional data centre (Data Linkage Queensland).

## Discussion

The PHRN has taken a distributed and flexible approach to the development of a national data linkage capacity. This approach has supported building data linkage capability in all jurisdictions as well as a cross-jurisdictional capability. A flexible approach allowed each jurisdiction to progress at their own pace and within the limitations of their legal and policy environment. At least initially, Australian data custodians were not comfortable copying all of their content data into central repositories. Attitudes have now changed and the majority of jurisdictions have repositories. This distributed and flexible approach has also meant that Australia has tackled the complex issues surrounding cross-jurisdictional linkage sooner than other multi-jurisdictional countries such as Canada and the UK who have both funded national initiatives recently [18, 19].

Although the distributed and flexible approach enabled Australia to shift from two jurisdictional data centres to data centres serving all Australian jurisdictions and a cross-jurisdictional capability, it has a number of challenges. Firstly, a distributed model is not very efficient as it requires multiple approvals and multiple data extractions for each approved research project. Secondly, linkage methods and content data formats are generally not harmonized between jurisdictions

The PHRN is addressing the need for improved efficiency in a number of ways including:

- Advocating for inclusion of research using linked data in the National Mutual Acceptance Scheme to reduce the number of ethics approvals required.
- Continuing to develop and improve the Online Application System and associated business processes and encourage jurisdictions to reduce the amount of information required in addition to that in the Online Application System.
- Planning for further development of repositories to reduce the time required for data extraction and improve data harmonization
- National Master Linkage Key for routine cross jurisdiction linkage
- Advocating for and supporting legislative change to reduce administrative burdens on researchers and data custodians.
- Supporting more flexible e.g. distributed computing options for conducting linkage to enable speedier and more efficient linkage and analysis.

To date there has not been much progress in harmonisation of data repositories and the development of associated metadata. This is an area that should be considered in the future. Some initial exploratory work has been conducted on linkage quality but more work is required to achieve standardised reporting and benchmarking.

## Conclusions

The PHRN is a national network of data centres which provides access to linked data on all Australians for researchers across Australia. Linked data is available from each individual Australian jurisdiction and this data can be pooled. Data can also be linked across Australian jurisdictions so data from an individual can be linked irrespective of their geographical location or the type of service they received.

Privacy and information security are of the highest priority for the PHRN. As the PHRN involves nine different jurisdictions all with different legal, regulation and policy requirements, each PHRN data centre implements a privacy by design approach appropriate for their jurisdictional environment. For cross-jurisdictional projects the jurisdictions work together to deliver the linked data required for the project using methods acceptable to each jurisdiction.

The PHRN’s flexible and distributed approach to data linkage has enabled the establishment of a truly national data linkage infrastructure. Continued development of the PHRN infrastructure will include a focus on improving the timeliness of access to linked data for researchers and the harmonisation of data repositories and associated metadata.
